# Recent Advances in Magnetic Microfluidic Biosensors

**DOI:** 10.3390/nano7070171

**Published:** 2017-07-06

**Authors:** Ioanna Giouroudi, Georgios Kokkinis

**Affiliations:** Institute of Sensor and Actuator Systems, Vienna University of Technology, Gusshausstrasse 27-29/366-MNS, Vienna 1040, Austria; georgios.kokkinis@tuwien.ac.at

**Keywords:** microfluidics, magnetic markers, magnetic biosensors

## Abstract

The development of portable biosening devices for the detection of biological entities such as biomolecules, pathogens, and cells has become extremely significant over the past years. Scientific research, driven by the promise for miniaturization and integration of complex laboratory equipment on inexpensive, reliable, and accurate devices, has successfully shifted several analytical and diagnostic methods to the submillimeter scale. The miniaturization process was made possible with the birth of microfluidics, a technology that could confine, manipulate, and mix very small volumes of liquids on devices integrated on standard silicon technology chips. Such devices are then directly translating the presence of these entities into an electronic signal that can be read out with a portable instrumentation. For the aforementioned tasks, the use of magnetic markers (magnetic particles—MPs—functionalized with ligands) in combination with the application of magnetic fields is being strongly investigated by research groups worldwide. The greatest merits of using magnetic fields are that they can be applied either externally or from integrated microconductors and they can be well-tuned by adjusting the applied current on the microconductors. Moreover, the magnetic markers can be manipulated inside microfluidic channels by high gradient magnetic fields that can in turn be detected by magnetic sensors. All the above make this technology an ideal candidate for the development of such microfluidic biosensors. In this review, focus is given only to very recent advances in biosensors that use microfluidics in combination with magnetic sensors and magnetic markers/nanoparticles.

## 1. Introduction

Biotechnological microsystems have drawn considerable attention during the last two decades; these systems are otherwise known as biochips, bioMEMS (microelectromechanical systems) or LOC (Lab-On-a-Chip) depending on the operations (e.g., analysis, detection, processing etc.) that are being integrated on a single microdevice. A biosensor, on the other hand, is a device used for the detection of pathogens or biomolecules and usually consists of: (i) a biological recognition component that interacts or reacts with the biological entity under investigation and (ii) a transducer that translates this recognition component into an electric output signal that can be measured with an appropriate instrumentation.

One of the major advantages of biotechnological microdevices is the reduction of costs of biological analysis due to limited fluid sample volume, multiplexing, and reduction of the quantities of reactants and the time needed to analyze a sample. Even the hazard of handling and detecting dangerous pathogens or chemicals is considered to be greatly reduced by the miniaturization of the reaction scale [1,2,3,4]. Additionally, their potential for integration with other analytical techniques offers a low-cost, portable solution. It is also expected that they can provide higher sensitivity than usual macroscopic techniques. For example, in disease cases—such as HIV or influenza—the method used to monitor and initiate treatment is a pathogen (e.g., viral) count of patients. In order to initiate treatment and regulate the dosage of therapeutic medication, and thus increase the survival rate, it is crucial to know the number of pathogens per unit volume of blood; these devices could have the potential of accurately providing such information.

The main asset of microfluidics over traditional benchtop analytical, chemical, and biomedical tools can be attributed to the unique behavior of fluids in the microscale; due to inherently small Reynolds numbers, fluids flow, almost exclusively within the laminar flow regime [5]. This allows for an accurate prediction and simulation of the flow profile. Equally important is that gravitational forces are negligible due to the small characteristic length of the devices, while surface and interfacial surface forces are dominant. These forces allow for a set of beneficial operations such as: passive pumping of fluids into the devices [6], user defined patterned surfaces [7], and filtering of unwanted substances. One additional advantage of microfluidics is the presence of the capillary forces. These permit the manipulation of fluids in narrow confinements and the counteracting of the gravitational forces. Most importantly, capillary forces can eliminate the need for pumps, integrated or not, simplifying the operation and the development of the system. The above-mentioned properties of microfluidics have been employed, in various approaches, in the design and development of the systems presented in this work.

Besides time and cost reduction, microfluidics enable the portability of the measurement instrumentation; essential tasks such as sample handling and analysis are being executed within the biochip and on-site without the demand of established laboratory infrastructure or well-trained personnel. In other words, the proposed microfluidic systems can lead to what is described as Point of Care (PoC) testing, namely diagnostic tests carried out in real-time and on-site. This revolutionary new practice leads to a shift of paradigm in traditional medicine, particularly towards a less centralized model, while improving diagnostics and biological research methods.

This progress is unquestionably beneficial for developing countries as they often lack access to temperature controlled laboratories and chemical storage rooms, expensive reagents, and highly trained personnel. The developed biotechnological microsystems substantially address these issues providing alternatives to traditional diagnostics and boosting the biomedical capacities of the developing world [8,9,10,11].

Well established optical and electrochemical techniques that have been used for biomedical diagnosis are not favorable for on-chip applications due to several technical challenges. Some of these challenges in the case of optical detection are: (i) photostability problems with time as well as narrow excitation range and broad emission spectra of the fluorescent labels [12,13,14,15] and (ii) the size and the cost of the measurement set-ups (fluorescent label excitation laser and optics for the detection).

Nevertheless, immunological tests (e.g., Enzyme-Linked Immunosorbent Assay (ELISA) immunoassays) and nucleic acid based diagnostics do not require a large amount of sample and provide rapid identification [16,17,18,19,20,21]. However, for the execution of such tests, established laboratory infrastructure and specialized personnel is either required or the fabrication of the test strips and the diagnostic devices is expensive and technologically complex. Furthermore, false positive and negative results may occur regardless of their sensitivity and specificity. Such results may be caused by improper washing or reagent deterioration or by improper storage or treatment of the sample. Thus, most of the reported microfluidic diagnostic platforms up to date either require complex on-chip designs, fluorescence or quantum dot labeling, nucleic acid amplification or continuous flow [22,23].

However, microfluidic diagnostic systems that use magnetic methods have provided an encouraging alternative for such portable robust devices [24,25,26,27,28,29]. This is due to the following facts: (i) magnetic fields can be applied externally or from microconductors that are directly integrated in the biosensing platform, (ii) magnetic fields can be well tuned by adjusting the applied current, (iii) the magnetic markers/nanoparticles used for the labeling of the biological entity can be manipulated inside microfluidic channels by high gradient magnetic fields that can in turn be detected by magnetic sensors, and (iv) the flexibility of the magnetic markers to label any entity due to functionalization by means of surface modification and specific binding. It is the multi-functionality of the magnetic markers that makes them ideal candidates as the active component in miniaturized on-chip biosensors.

**Figure 1:**
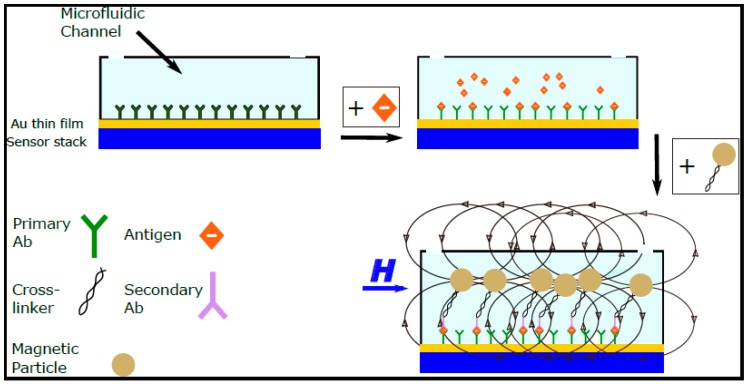
Schematic of the working principle of a standard magnetic biosensor.

Moreover, the magnetic sensors used for the detection of the markers’ stray field are compatible with standard silicon integrated circuit (IC) technology, and thus suitable for integration into hand held, portable on-chip biosensing systems. Figure 1 shows a schematic of the working principle of the most commonly developed magnetic biosensors which are based on the “sandwich” method; the surface of the sensor is functionalized with ligands and the sample under investigation is mixed with the functionalized magnetic particles externally and then inserted in the microfluidic channel above the sensor’s surface. Magnetic biosensors have emerged as excellent pathogen detection devices at room temperature and as quantification methods of biological entities due to their high sensitivity, less complex instrumentation, compact size, and integration flexibility. Current efforts are focused on the integration of such sensors within microfluidic platforms to develop simple, sensitive, and portable devices for rapid diagnosis of diseases [30].

While a detailed description of the theory, fabrication, operation, and application of magnetic microfludic biosensors has been presented in several review articles [31,32] in the past, this review focuses only on the advances in this field that were made in the last couple of years and specifically on techniques that combine magnetic labeling, microfluidics, and magnetic sensing. A short description of the methodologies, developed devices, and results of each reviewed research work will be presented.

## 2. Magnetic Microfluidic Biosensors: State of the Art

Researchers from the University of Minnesota [33] developed a sensitive, giant magnetoresistance (GMR) biosensor for the simple detection of influenza A virus. The developed assay uses monoclonal antibodies to viral nucleoprotein (NP) combined with magnetic nanoparticles (MNPs). It is a so-called “sandwich” method where the Influenza virus, when present, permits the binding of the MNPs to the GMR sensor and this binding is proportional to the concentration of the virus. A change in the resistance of the sensor is caused by the binding of the MNPs onto the GMR sensor surface, which is then measured in a real time electrical readout.

Technologies like the Shamrock Magnetron Sputter System, photolithography, ion beam milling, and electron beam evaporation were employed for the fabrication of the GMR chips. The sensor surface was functionalized with aldehyde groups. These allow subsequent covalent bonding of biomolecules that contain amino groups onto the GMR sensor. A bottomless reaction well fabricated by polymethylmethacrylate (PMMA) was attached onto the chip and centered at the sensor area. All details on the test preparation, the influenza A virus immunoassay and the enzyme linked immunosorbent assay can be found in [33].

The obtained results indicated that the developed GMR biosensor is able to detect virus concentrations ranging from 1.5 × 10^2^ TCID_50_/mL to 1.0 × 10^5^ TCID_50_/mL. According to the medical dictionary; TCID_50_ is the median tissue culture infective dose. It is the amount of a pathogenic agent that will produce pathological change in 50% of cell cultures inoculated. Expressed as TCID_50_/mL. These results showed that their GMR biosensor assay could be potentially applied for diagnosis and monitoring of influenza virus since the virus concentration in nasal samples of influenza virus infected swine was reported to be in the range of 10^3^ to 10^5^ TCID_50_/mL [33].

The authors of [34] reported in 2016 on the detection of magnetically labeled DNA by means of a disposable card (SPA) and an anisotropic magnetoresistive (AMR) sensor. The magnetically labeled DNA was immobilized on a disposable card. The SPA was fabricated as follows; a polydimethylsiloxane (PDMS) thin layer was first deposited by spin coating on silicon substrate, followed by ultraviolet/ozone (UVO) treatment, 3-aminopropyltriethoxy (APTES) grafting, functionalizing using succinic acid anhydride (SAA), and SPA probe immobilizing. The AMR sensor consisted of Wheatstone bridges incorporating a serially connected ensemble of simple AMR elements of 5 nm thick Ni_80_Fe_20_ film. The film was grown on SiO_2_/Si by using magnetron sputtering equipment (model ATC 2000) and structured by UV lithography. The DNA was magnetically labeled by superparamagnetic 1 μm Dynabeads^®^MyOne™ streptavidin C1 (with 26% ferrites) markers. For the measurements the SPA card was placed at a distance of 10 μm from the sensor surface.

The researchers identified an almost linear response between the output signal of the AMR sensor and the amount of single-stranded target DNA varying from 4.5 to 18 pmol. From the sensor output signal response towards the mass of the magnetic markers, which were directly immobilized on the disposable card surface, the limit of detection was estimated to be about 312 ng ferrites, which corresponds to 3.8 μemu. Their approach opens up new possibilities for the development of highly sensitive, low-cost, and rapid-detection DNA biosensors.

**Figure 2:**
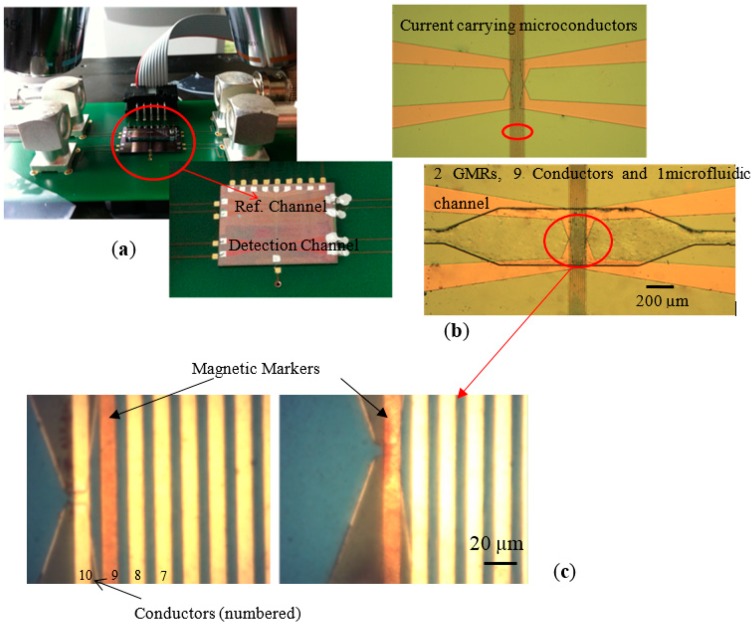
(**a**) The developed biosensor that consists of the giant magnetoresistance (GMR) sensors, the microconductors and the microfluidic channels; (**b**) Microscope image of the microconductors and a microfluidic channel; (**c**) Detailed image of the microconductors and the GMR sensor with magnetic markers as they moved from the right to the left microconductor.

In 2016 researchers from the Vienna University of Technology developed a versatile, dual-purpose GMR biosensor for in vitro detection of Enterobacteriaceae (e.g., *Escherichia coli*) and biotinylated antibodies (e.g., IgG rabbit polyclonal antibodies) as shown in Figure 2 [35].

These biological entities are labeled with functionalized MNPs, suspended into a static fluid and injected by a syringe into a microfluidic channel (detection channel). The labeled bioanalytes (pathogens and antibodies) are set in motion by a magnetic force applied by integrated microconductors. Bulk or complicated pumping systems are not required. The novel detection principle is based on the velocity decrease of the MNPs that are loaded with the respective bioanalyte due to factors inhibiting their motion. The velocity of unloaded, bare markers injected by a syringe in another, parallel microfluidic channel is used as a reference.

The authors of [35] discovered that the constrained particle motion is based on the following: (i) in the case of *E. coli*, the inhibiting factor is the enhanced Stokes’ drag force due to the greater volume and altered hydrodynamic shape of the MNPs, whereas (ii) the case of biotinylated antibodies, it is the increased friction force at the interface between the modified MNP and the biosensor’s surface. Their biosensing method differs from the standard ones as it does not involve a sandwich structure for detection. For the first time, friction force is used in a scheme for resolving biomolecules. The velocity is measured by means of integrated magnetic GMR sensors that detect the MNPs’ stray field and are positioned underneath the inlet and outlet of the microfluidic channel. The authors also developed a surface modification that is biocompatible, easy to implement and reliable, and practically diminishes the problem of bioadhesion on the sensor’s surface.

**Figure 3:**
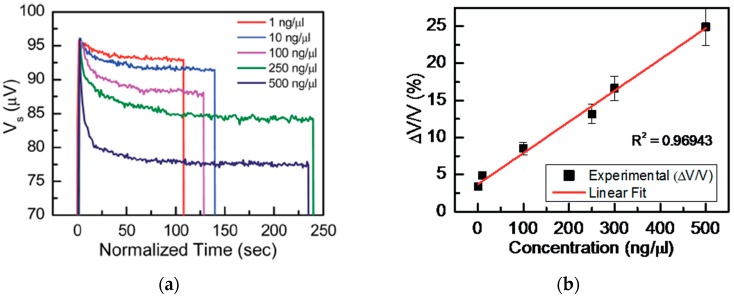
(**a**) Change in the sensor voltage with accumulation of superparamagnetic Nanomag-D beads on the micro-conductor with respect to the normalized time (*t* assumed zero at the beginning of the measurement); (**b**) Relative change in the voltage across the GMR sensor head due to the presence of various concentrations of Nanomag-D beads on the microconductor (MC) (Reproduced with permission from [36]. The Royal Society of Chemistry, 2015.).

Moreover, in order to demonstrate the application of a spin valve GMR integrated microfluidic sensor for the detection and quantification of superparamagnetic nanomarkers various concentrations of Nanomag-D beads of 250 nm diameter were tested by the authors [36]. The results show that the sensor is capable of quantifying concentrations of Nanomag-D beads in a linear scale over a wide particle concentration range (1–500 ng·µL^−1^). Specifically, Figure 3a shows the sensor voltage *V*_s_ as a function of time (*t*) measured for various concentrations in the range of 1 ng·µL^−1^ to 500 ng·µL^−1^ of the magnetic nanobeads.

From a biosensing perspective, a good biosensor should be capable of detecting low particle concentrations and effectively quantifying particle concentrations over a large and linear scale. Therefore, the authors also calculated the relative change in voltage for various concentrations of Nanomag-D beads as reported in [36], which can be used to tag biomolecules when functionalized. The calculated results and their linear fits are shown in Figure 3b.

Another interesting biosensing platform was reported in [37] for rapid high-precision quantitative analyses for in vitro diagnostics. The platform combines the merits of sandwich immunochromatography with highly sensitive quantification of 200 nm MNPs from the entire volume of lateral flow membranes. Specifically, test strips are passed through a measuring coil of a portable magnetic particle quantification (MPQ) reader to quantify the number of MNPs bound at the test line (TL) and control line (CL). In short, the MPQ technique employs a non-linear magnetization of MNPs subjected to a magnetic field at AC frequencies *f*_1_ and *f*_2_ by recording the MNP response at a combinatorial frequency *f* = *n*∙*f*_1_ + *m*∙*f*_2_, where *n* and *m* are integers (one of them can be zero). The method is insensitive to linear dia- and paramagnetic materials. Additionally, its sensitivity to magneto-radioactive ^59^Fe-based MP is on the level of the γ-radioactive technique.

The performance of the platform was exhibited by quantitatively measuring concentration of a disease marker of prostate-specific antigen (PSA) in human serum of as low as 25 pg/mL total PSA during a 30-min assay.

The featured three-fold signal increase per every order of concentration within 3.5 orders of magnitude permits an actual analysis of antigen concentrations in a wide range. Some possible applications of this tool are: (i) quick and convenient optimization of IC test systems, (ii) thorough control of assay parameters, and (iii) enhancement of assay accuracy.

**Figure 4:**
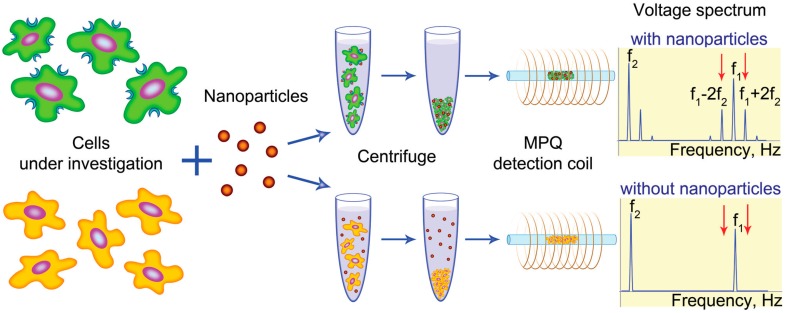
Schematic of the magnetic particle quantification (MPQ)-cytometry workflow. The investigated cells are incubated with the suspended nanoparticles. The unbound nanoparticles are then removed from the sample by centrifugation. The amount of nanoparticles bound to the cells is measured using the magnetic particle quantification (MPQ) technique at combinatorial frequencies (Reproduced with permission from [38]. The Royal Society of Chemistry, 2016.).

Another rapid method for studying the surface of cells using biomolecules on MNPs using the MPQ technique, called MPQ-cytometry, was demonstrated in [38]. Specifically, it is the integral non-linear response produced by magnetically labeled biomolecules in a cell sample that is measured with the MPQ technique (see Figure 4). The authors proved that the new method allows a rapid assessment of the oncological HER2/neu status of cells and confirmed high correlation of the obtained data with the data of the standard labor-intensive methods of cytometry.

The developed method allows one to detect pathogenic cells in complex non-transparent media, as well as to estimate the expression level of various antigens on the cell surface. MPQ-cytometry provides a sensitivity limit of 0.33 ng of MNPs and is lacking background signal otherwise present in many label-based assays. Each measurement lasts only a few seconds, and complicated sample preparation or data processing is not necessary.

**Figure 5:**
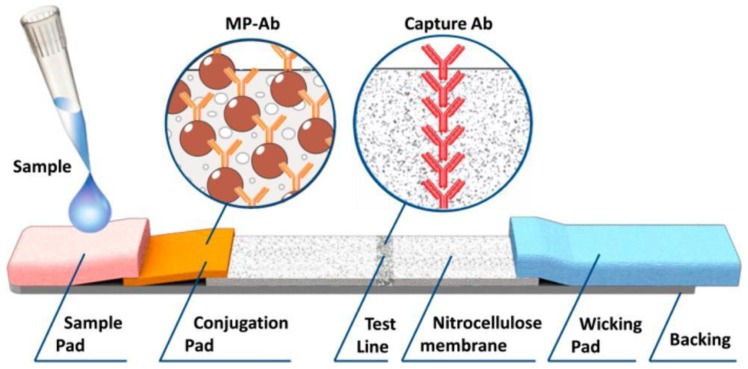
Schematic of a test strip based on sandwich-lateral flow assay with antibody-conjugated magnetic particles as labels (Reproduced with permission from [39]. ACS Publications, 2016.).

A multiplex quantitative lateral flow (LF) assay for simultaneous on-site detection of several antigens in complex matrixes was developed by the authors of [39] and published in Analytical Chemistry in 2016. The developed assay is innovative by virtually no sacrifice in performance while transitioning from the single-plex assays and by having characteristics on the level of laboratory quantitative methods. The novel approach to easy multiplexing is realized via joining an on-demand set of single-plex LF strips, which employ magnetic nanolabels in a miniature cylinder cartridge that mimics LF strips during all assay stages. The cartridge is read out by an original portable multichannel reader based again on the above mentioned MPQ technique. The proposed assay is based on the sandwich lateral flow assay principle with MP labels [40]. A test sample is deposited onto the sample pad and under capillary action the sample migrates along the strip and interacts with dry MP-Ab conjugates deposited at the conjugation pad. Then, if the target antigen is contained in the sample, it binds to MP-Ab and to the capture Ab on the test line (TL) (see Figure 5).

The developed multichannel reader offers the unmatched 60 zmol detection limit and seven-order linear dynamic range for volumetric registration of magnetic labels inside a cartridge of several millimeters in diameter, regardless of its optical transparency.

The multiplex assay performance was successfully validated in [39] by botulinum neurotoxin (BoNT) types A, B, and E detection in milk and apple and orange juices. The developed method can be extended to other proteins and used for rapid multianalyte tests for point-of-care in vitro diagnostics, food analysis, biosafety and environmental monitoring, forensics, and security, etc.

In 2014, the authors of [41] used the magneto-reactance (MX) effect of a Co_65_Fe_4_Ni_2_Si_15_B_14_ amorphous ribbon with a nanohole-patterned surface to develop a reliable, biocompatible biosensor. Curcumin labeled to superparamagnetic (Fe_3_O_4_) nanoparticles was detected and quantified by means of the novel biosensor. The change in MX of the ribbon subject to varying concentrations of the Fe_3_O_4_ nanoparticles to which Curcumin was attached was used to assess the detection and quantification of Curcumin. The authors of [41] achieved quantitative analysis of Curcumin-loaded Fe_3_O_4_ nanoparticles in the range of 0–50 ng/mL, beyond which the detection sensitivity of the sensor remained unchanged. A 30% high detection sensitivity, which is about 4–5 times higher than that of a magneto-impedance (MI) based biosensor, proves the high capacity of the MX-based biosensor. A possible application of the developed biosensor could be the detection of low-concentration magnetic biomarkers in biological systems.

Recently, authors from the INESC-MN Institute in Portugal reported a microfluidic device for the detection of *Streptococcus agalactiae* (a Group B streptococci) and *Streptococcus uberis* in raw milk samples [42]. The device, pointing towards a POC system for mastitis diagnosis in dairy farms, uses iron oxide MNPs as immunomagnetic agents. The MNPs, linked with antibodies, capture the targeted cells. As the sample flows through the microfluidic device, an array of spin valves detects the particles clustered around the targeted cells and peaks appear at the sensor’s outlet. The authors reported a lower detection limit of 100 cfu/mL, independently of targeted bacteria and antibodies. The experiments were cross-referenced with the PCR method, the method exhibits a 73% positive detection in samples with streptococci species using an *anti-S. agalactiae* antibody, and 41% using an anti-GB streptococci antibody.

An equal lower detection limit (100 cfu/mL) was reported by the authors of [43]. Similarly to the previous approach, the device used GMR sensors and antibody tagged particles (Dynabeads^®^). Instead of a magnetic cytometry implementation, the authors used an ELISA approach, where the antigen was captured between two antibodies; one immobilized on the substrate and the second attached to a magnetic marker. The magnetic marker provided the signal upon which the detection is based. The described biosensor was tested with *Escherichia coli* (*E. coli*) O157H:H7.

Lastly, magnetic traps and TMR (tunneling magnetoresistance) magnetic sensors for the entrapment and detection of magnetic labels were demonstrated in [44]. The authors used on-chip conductive structures, which upon sequential current supply realized a focusing of the magnetic labels (surface modified magnetic particles) over the sensing area. Thus, even small concentrations of magnetic particles with their corresponding analyte can be detected. The authors optimized crucial parameters for the biosensor’s operation, while experiments with different commercial magnetic markers were conducted.

## 3. Conclusions

In conclusion, microfluidics can be the foundation that leads to the development of new devices to be applied in the fields of life sciences, biotechnology, pharmaceuticals, and agriculture. The fact that many chemical, biological, and biophysical processes and tasks are carried out in liquid environments allows the employment of such microfluidic platforms for biomedical analysis in a portable, reliable, and simple manner.

The presented biosensing systems offer compact, highly-sensitive, and cost-effective solutions for biomedical diagnostics. Through our study, it was concluded that several novel detection mechanisms combining magnetic markers, magnetic sensors, and microfluidics are continuously emerging. Therefore, magnetic biosensors could in fact become competitive counterparts and even replace in the future the currently available optical and fluorescence detection systems, while maintaining high specificity, sensitivity, and fast readout times. Compared to such standard technologies, magnetic microfluidic biosensors directly translate the presence of a biological entity into an electrical signal, thus providing a fully electronic readout and enabling the future development of point-of-care devices.
